# A multilevel adaptation approach for the sustainable scale-up of family cancer history screening in rural public health clinics

**DOI:** 10.1186/s13690-026-02018-7

**Published:** 2026-07-17

**Authors:** Yue Guan, Shacoria Anderson, Cindy Snyder, Angela Zhang, Cynthia Walters, Dayanna Ramirez Leon, Colleen M. McBride, Yuan Liu, Sheryl Gabram, Denise Ballard, Joseph Lipscomb, Lauren E. McCullough, Cam Escoffery

**Affiliations:** 1 Department of Behavioral, Social and Health Education Sciences, Rollins School of Public Health, Emory University, 1518 Clifton Road, NE, Atlanta, GA 30322 USA; 2 Georgia Center for Oncology Research and Education, Atlanta, GA USA; 3 Georgia Department of Public Health, Southwest Health, District 8-2, Albany, GA USA; 4 Department of Biostatistics and Bioinformatics, Rollins School of Public Health, Emory University, Atlanta, GA USA; 5 Department of Health Policy and Management, Rollins School of Public Health, Emory University, Atlanta, GA USA; 6 Department of Epidemiology, Rollins School of Public Health, Emory University, Atlanta, GA USA; 7 Winship Cancer Institute, Emory University, Atlanta, GA USA

**Keywords:** Family cancer history screening, Adaptation, Hereditary cancers, Rural health, Multilevel

## Abstract

**Background:**

National guidelines (e.g., the U.S. Preventive Services Task Force) and public health organizations (e.g., the Centers for Disease Control and Prevention) recommend population-level family cancer history screening to enhance hereditary cancer risk assessment. Despite over a decade of statewide implementation in Georgia, participation in and the impact of family cancer history screening in rural public health clinics remains limited, highlighting the need for systematic adaptation of existing program delivery approaches. Furthermore, the application of implementation frameworks to the documentation and evaluation of adaptations in genomic service delivery is infrequent. In this report, we apply the Exploration, Preparation, Implementation, and Sustainment (EPIS) framework and the Framework for Reporting Adaptations and Modifications-Enhanced (FRAME) to systematically document multilevel adaptations aimed to increase access to Georgia’s statewide family cancer history screening program for *BRCA*-associated cancers among women in rural Georgia.

**Methods:**

We conducted a 14-month adaptation process in the Southwest Public Health District of Georgia from November 2023 to January 2025. Using the FRAME, we systematically documented adaptations in five steps: (1) stakeholder identification, (2) multilevel needs assessment, (3) development of prototype adaptations, (4) adaptation validation and prioritization through stakeholder deliberation, and (5) pilot testing.

**Results:**

We convened a stakeholder committee (*n* = 15) representing multiple levels of healthcare delivery, including patients, internal stakeholders from 14 public health clinics in the district, and external stakeholders from regional cancer coalitions and healthcare systems. A total of 15 adaptations were prioritized across individual (*n* = 2), provider (*n* = 1), clinic (*n* = 7), organization (*n* = 3), and community (*n* = 2) levels. Most adaptations focused on implementation and scale-up activities (*n* = 10), followed by changes to contextual modifications (*n* = 4) and communication content (*n* = 1). Key adaptations included new outreach (e.g., posters, phone scripts, social media), streamlined screening workflow (e.g., simplified data entry, automated referrals), and integration of theory-based, risk-stratified communications about screening results.

**Conclusions:**

Strong partnerships between public health agencies, community organizations, and academic institutions were essential to guiding our adaptation process. By combining implementation frameworks, multilevel stakeholder engagement, and proactive pre-implementation adaptation, our study offers a replicable, stepwise approach for adapting, scaling up, and sustaining other hereditary cancer screening programs in underserved public health settings.

**Supplementary Information:**

The online version contains supplementary material available at https://doi.org/10.1186/s13690-026-02018-7.

**Table:** 

Text box 1. Contributions to the literature
∙ This study demonstrates how implementation science frameworks can be operationalized to systematically guide and document multilevel adaptations in public health clinics.
∙ Our approach highlights the value of engaging multilevel stakeholders to shape program priorities, enhance contextual fit, and address barriers in healthcare settings.
∙ We provide a replicable, stepwise approach to inform the adaptation, scaling up, and sustainment of other hereditary cancer screening programs in underserved public health settings.

## Background

### Family cancer history screening for BRCA-associated cancers

National guidelines and tiered ratings of genomics applications suitable for population use have been available for more than a decade [1], yet population-level implementation has lagged despite rapid advances in health-related genomic discovery. Systematic population-based implementation of family cancer history screening is a critical step toward identifying individuals at increased genetic risk for *BRCA*-associated cancers [2–4]. Women carrying *BRCA1/2* mutations face significantly increased lifetime risks for breast (50–80%) and ovarian (10–40%) cancers, and benefit from evidence-based preventive interventions, including enhanced screening, chemoprevention, and prophylactic surgeries [5, 6]. The development of brief, validated screening tools for hereditary cancer risk—such as the Ontario Family History Assessment Tool (FHAT) [7]—endorsed by national guidelines (e.g., U.S. Preventive Services Task Force [USPSTF]) and public health agencies (e.g., Centers for Disease Control and Prevention [CDC] ) has enabled genetic risk assessment at the population level.

### Population-based hereditary cancer screening in public health settings

Statewide implementation of such screening in rural public health settings offers a promising opportunity to reduce disparities in access to genomic services [8]. Since 2012, Georgia has led a statewide initiative to integrate family cancer history screening into its public health infrastructure, with the Department of Public Health (DPH) administering the Georgia Center for Oncology Research and Education (CORE)’s screening program across public health clinics. The majority of women who visit these clinics are uninsured and live below 200% of the federal poverty level. Women with scheduled visits at participating public health clinics are approached by a nurse who completes a genetic risk assessment using the FHAT. The FHAT assigns points based on the number of first, second, and/or third-degree relatives diagnosed with breast, ovarian, colon, or prostate cancer, as well as age at diagnosis and the type and number of primary cancers. Patients with scores of 10 or higher meet the referral threshold for genetic counseling [7]. The FHAT has a sensitivity of 91–94% and a specificity of 15–51% for identifying individuals at increased risk [7, 9–11]. FHAT scores categorize patients into two *BRCA*-associated cancer risk groups: “positive” (high risk for *BRCA1/2* mutation; genetic counseling recommended) and “negative” (unlikely to carry a *BRCA1/2* mutation). FHAT scoring is used to guide referrals for genetic counseling, with historical data indicating that 4–6% of screened individuals meet high-risk criteria [12].

Despite more than a decade of statewide implementation, participation in and the impact of Georgia CORE’s family cancer history screening program remain limited. We conducted an eight-year evaluation of Georgia CORE’s screening program to assess program reach and women’s uptake of cancer genetic services [12]. Between 2012 and 2020, the program was offered in 81 out of 187 (43%) public health clinics in Georgia, reaching women in 75 of 159 (47%) counties statewide, including 69 counties that are considered as medically underserved areas. To date, more than 30,000 women have completed family cancer history screening across Georgia, with 57% self-identifying as a member of racial/ethnic minority groups. However, this geographic distribution did not translate into meaningful population-level participation, as fewer than 5% of age-eligible women completed screening, and only 35% of those meeting high-risk criteria received genetic testing [12].

To further understand community perspectives, our team collaborated with community-based networks in Southwest Georgia to engage residents of African Ancestry (*N* = 24) in a structured community discussion in which residents weighed the benefits and potential concerns of expanding Georgia CORE’s family history screening program for *BRCA*-associated cancers. Participants reached consensus that public health clinics are highly credible sources of primary care for rural women and viewed the current program as important for increasing access to life-saving options among women at increased risk for breast cancer [13]. However, participants’ awareness of the screening program was low (25%), with the vast majority strongly endorsing that it should be expanded (96%) and calling for improved screening outreach and result communication. Taken together, these findings highlight the need for additional community assessment and program adaptation to increase the number of women screened and improve the effectiveness of screening result communications in rural Georgia, where large populations of ethnic minorities reside.

### Addressing program reach and sustainability through adaptation

Addressing these gaps in screening uptake and resulting communication requires strategic adaptation of existing clinic-based approaches within public health settings. Adaptation is defined as the deliberate modification of an evidence-based intervention to meet the needs of target populations and community contexts while preserving its core components [14]. With funding from the National Cancer Institute and in partnership with the Georgia Department of Public Health and community organizations in Southwest Georgia, we conducted a multilevel adaptation of Georgia CORE’s statewide family cancer history screening program. The goal was to increase the number of women screened and improve access to risk-stratified services. When adapting programs for public health settings, researchers must consider resource constraints, workflow changes, shifts in staff behavior or attitudes, and decision-making that operate across multiple levels. At the same time, systematic documentation of what adaptations are made, how they are implemented, and when they occur is essential to evaluate both implementation and effectiveness outcomes [15, 16]. This is especially important in population-based family history screening programs, where the intervention and delivery context involve multiple layers of complexity.

Despite decades of availability, implementation frameworks to support the dissemination of evidence-based interventions have not been widely applied in genomic contexts [17, 18]. Frameworks such as the Reach, Effectiveness, Adoption, Implementation, and Maintenance (RE-AIM) and the Consolidated Framework for Implementation Research (CFIR) have been used to evaluate factors influencing the implementation of evidence-based interventions [19]. However, a critical gap remains in the systematic reporting of adaptations and implementation strategies necessary to integrate interventions into real-world settings. The Framework for Reporting Adaptations and Modifications-Enhanced (FRAME), developed by Stirman et al. [20], offers a pragmatic, theory-informed structure for systematically characterizing adaptations across levels. Although FRAME is increasingly used in implementation science research [16], its application to genomic service delivery remains limited. One study used a modified version of FRAME to track adaptations made during the implementation phase of a population-based genetic screening program focused on high-priority (Tier 1) conditions identified by the CDC [21]. However, no prior studies have applied this framework to document adaptations occurring during the pre-implementation phase. This represents a missed opportunity, as adaptations made during pre-implementation may be more effective than reactive modifications introduced during implementation [22].

This study applies the FRAME to examine and report the multilevel adaptation process for Georgia’s statewide family cancer history screening program, extending prior research and offering a case example of proactive adaptations in population-based hereditary cancer screening programs.

## Methods

### Description of the parent project

**Fig. 1:**
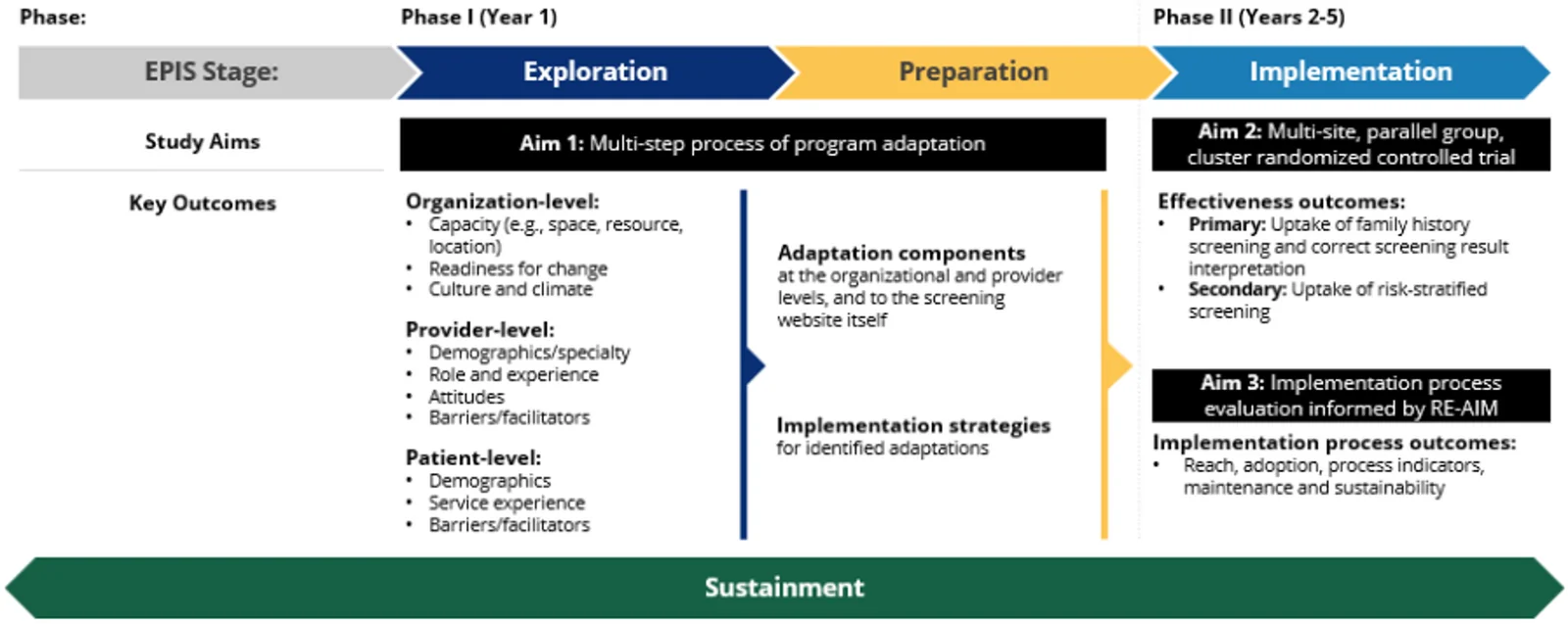
Study design for a family cancer history screening program adaptation project, informed by the Exploration, Preparation, Implementation, and Sustainment (EPIS) framework, Georgia, United States, 2023–2027. Abbreviations: EPIS, Exploration, Preparation, Implementation, Sustainment; RE-AIM, Reach, Effectiveness, Adoption, Implementation, and Maintenance

The parent NCI-funded R37 project aims to increase the uptake and sustainability of Georgia CORE’s family cancer history screening program using the Exploration, Preparation, Implementation and Sustainment (EPIS) framework [23]. EPIS provides a multilevel, stakeholder-engaged adaptation process that involves four successive stages, with long-term program impacts (i.e., sustainment) considered throughout its lifecycle. Our project phases, aims, and key outcomes were designed to align with EPIS stages (Fig. 1). The current manuscript reports findings from Phase 1 only (Exploration and Preparation stages), which comprised 14 months of stakeholder engagement in Georgia’s Southwest Public Health District from November 2023 to January 2025. This phase involved a multilevel assessment of organizational, community, provider- and patient-level factors to identify barriers and facilitators to screening activities, characterize service gaps, and inform program adaptations and implementation strategies. Phase 2 (Implementation stage), outside the scope of this manuscript, is a cluster randomized controlled trial evaluating the adapted program across six public health clinics (3 intervention, 3 standard care) in the Southwest Public Health District. The intervention was launched in February 2025, with data collection ongoing through February 2028.

### Adaptation process description

**Fig. 2:**
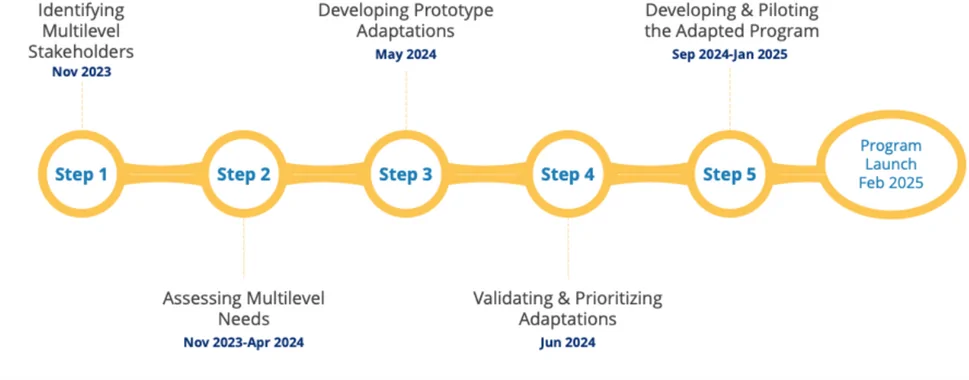
Flow chart of adaptations to the family cancer history screening program, Georgia, United States, 2023–2025

During Phase 1, adaptation followed five steps: [1] identifying stakeholders across multiple levels; [2] conducting a multilevel assessment of organizational, provider, and patient factors to identify barriers, facilitators, service gaps, and adaptation needs; [3] developing a prototype of recommended adaptations; [4] validating and prioritizing the prototype with stakeholders; and [5] developing and pilot-testing program adaptations and implementation strategies (Fig. 2).

Adaptations were coded using the FRAME framework, including the specific modification made, the nature of the modification (e.g., adding, removing, or expanding components), the level at which the modification occurred (e.g., organizational, community, provider, or patient), the goal and rationale for the modification, the individuals involved in the decision-making process, and whether the modification was fidelity-consistent [20, 24]. Fidelity-consistent adaptations preserve the program’s core functions while maintaining its effectiveness in promoting family cancer history screening. As this study focused on pre-implementation adaptations, all modifications were proactive and planned in response to identified barriers and gaps. Reporting followed the Standards for Reporting Implementation Studies (StaRI) guidelines [24].

### Step 1. Identifying multilevel stakeholders

**Fig. 3:**
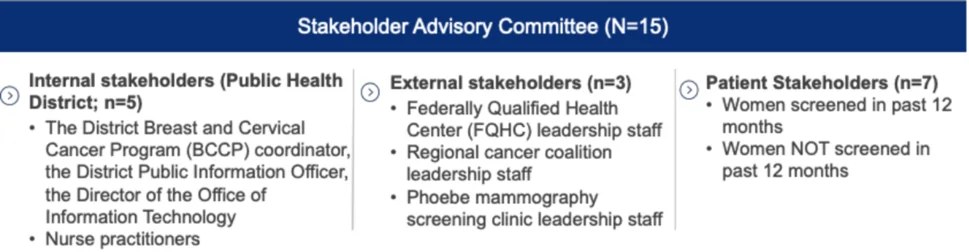
Multilevel stakeholder composition for a family cancer history screening program adaptation project, Georgia, United States, 2023

A stakeholder committee (*n* = 15) was convened to represent multiple levels of healthcare delivery involved in the family cancer history screening process (Fig. 3). *Internal stakeholders* (*n* = 5) were invited from the 14 public health clinics in Georgia’s Southwest District and included nurse practitioners, the District Breast and Cervical Cancer Program (BCCP) coordinator, the District Public Information Officer, and the Director of the Office of Information Technology. *External stakeholders* (*n* = 3) represented local organizations across the screening continuum, ranging from upstream community-based organizations to downstream service providers, ensuring consideration of care coordination and follow-up services. *Patient stakeholders* (*n* = 7) were women aged 25 and older who received primary care in the Southwest Public Health District, including both those who had completed screening within the past 12 months and those who had not.

### Step 2. Assessing multilevel needs

A comprehensive, multilevel needs assessment was conducted to identify implementation barriers and facilitators across individual, provider, and organizational levels. This assessment involved workflow analysis, observations, and interviews.

#### Workflow assessment

Between November and December 2023, we conducted semi-structured interviews with clinic leadership (e.g., medical directors, nurse managers) and frontline staff (e.g., registered nurses, nurse practitioners) at six public health clinics (see Supplemental File for interview guides). Participants described workflows for scheduling, screening administration, result communication, and follow-up coordination. Site-specific workflow diagrams were developed and synthesized into a master diagram representing district-wide workflow.

#### Prospective observations

In December 2023, we conducted a one-month “time study” at the six participating clinics where providers recorded patient volume and characteristics, time spent on key screening activities, and workflow challenges or patient concerns. In February 2024, the study team conducted a site visit to gain in-depth insight into contextual factors influencing screening delivery. Findings from the time study and site visit informed refinement of the master workflow diagram and contextualized implementation barriers and facilitators.

#### Internal stakeholder interviews

Using the master workflow diagram as a reference, we interviewed 11 internal stakeholders in April 2024 to identify implementation gaps, unintended consequences, and potential modifications to improve screening delivery and sustainability. 

### Step 3. Developing prototype adaptations

In May 2024, the study team conducted a rapid qualitative analysis [25] of interview data from Step 2 to identify multilevel implementation barriers and facilitators. Results informed the development of a prototype adaptation workbook (*Supplemental File for stakeholder workbook*) summarizing the current screening workflow and highlighting potential adaptation strategies targeting screening scheduling, delivery, and follow-up. The workbook was used to facilitate stakeholder deliberation in Step 4.

### Step 4. Validating and prioritizing adaptations

To validate and prioritize the adaptations identified in Step 3, the stakeholder committee participated in a 90-minute virtual deliberation session to validate and prioritize the proposed adaptations in June 2024. Prior to each session, stakeholders were asked to review the prototype adaptation workbook. During the session, participants completed a live poll to select their top three strategies for key steps in the screening process. Next, participants were divided into facilitated small groups (Group 1: internal and external stakeholders; Group 2: patients) to discuss the poll results before reconvening for a large-group discussion to establish strategy priorities. Effective deliberation requires participants to discuss and weigh differing, and often competing, social values to reach group consensus [13, 26]. Discussion facilitators received training from a deliberative democracy expert (MCG), with a focus on applying core deliberation principles. These included ensuring equal participation by providing all participants with the opportunity to contribute, encouraging reasoned justification by asking participants to reflect on others’ perspectives and articulate the rationale for their views, and promoting a societal perspective that emphasized what would best serve the community as a whole rather than their individual interests.

Three days later, participants completed a follow-up survey rating each strategy on feasibility, sustainability, effectiveness, and acceptability. For example, one survey item asked: “Thinking about how easy, doable, effective, and long-lasting these strategies are, which strategies do you think would most increase the number of women engaged?” Descriptive analyses were conducted on quantitative survey responses for each strategy. Responses were stratified by stakeholder category (i.e., internal/external stakeholders vs. patients) to identify areas of agreement and divergence.

### Step 5. Developing and pilot testing the adapted program

Following stakeholder prioritization, the study team finalized program adaptations by assessing each proposed strategy for feasibility, acceptability, effectiveness, sustainability, and alignment with DPH systems and policies. The team then developed intervention materials and, in collaboration with Georgia CORE’s website developer and the DPH information technology (IT) team, adapted the family cancer history screening website and implemented corresponding modifications within the DPH electronic medical record (EMR) system. Usability testing was conducted between December 2024 and January 2025 with study team members, clinic providers, and patients to refine intervention materials and delivery processes.

## Results

### Workflow of established family cancer history screening programs

**Fig. 4:**
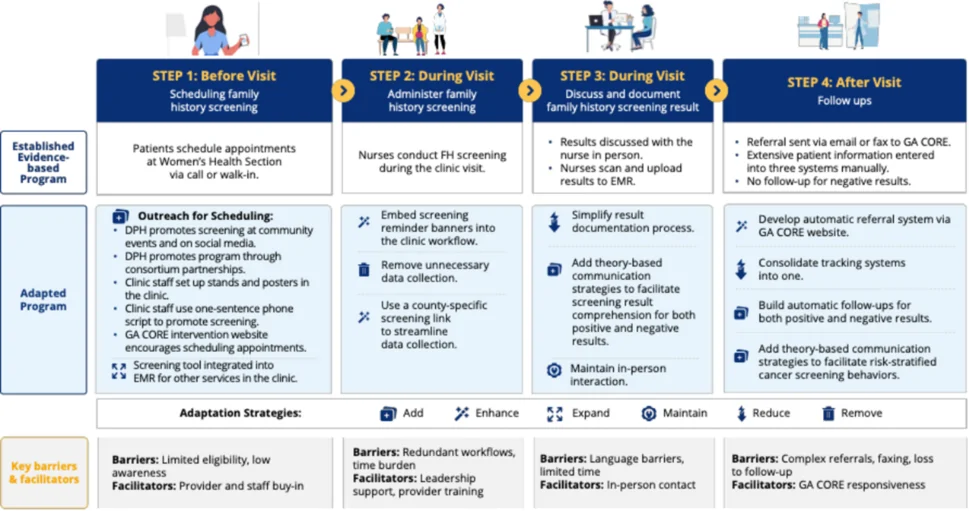
Comparison of established and adapted family cancer history screening program workflow, strategies, and implementation barriers and facilitators in rural public health clinics, Georgia, United States, 2023–2025

Workflow analysis identified four key steps in the existing family cancer history screening process *(*Fig. 4). Patients typically schedule an annual Women’s Health visit either by phone or as a walk-in. During the visit, a nurse administers the FHAT via the Georgia CORE website. The screening tool takes approximately 5 min to complete (range: 2–10 min). Explaining a positive result requires additional time (average = 15 min, range: 5–20 min), whereas negative results are usually not discussed in detail.

For patients with positive screening results, the nurse completes the Georgia CORE referral form, updates the patient’s chart, and faxes the referral to the Georgia CORE Advanced Clinical Genomics Nurse (ACGN) for further assessment. If the patient is eligible for genetic testing, the ACGN coordinates saliva or blood sample collection and communicates test results to both the patient and referring providers. For patients with negative screening results, there is no follow-up beyond routine updates to family cancer history during the next annual visit.

### Barriers and facilitators of screening program implementation

The needs assessment identified barriers and facilitators at each step of the family cancer history screening workflow. During appointment scheduling, key barriers included the limited eligibility among women attending in-person Women’s Health visits and the requirement to complete screening during the same visit. A major facilitator at this stage was the positive attitude of providers and staff toward screening. For screening administration, workflow redundancies were the primary barrier, while district- and clinic-level endorsement and provider training supported implementation. In the communication of screening results, language barriers and limited time for explanation were identified as significant challenges. Finally, for referral and follow-up, barriers included a multi-step referral process, reliance on faxing, and patient loss to follow-up, whereas Georgia CORE’s responsiveness was noted as an important facilitator.

### Prioritization of adaptation strategies

Internal stakeholders first reviewed the existing family history screening workflow and identified 23 specific gaps and 54 corresponding adaptation strategies (*Appendix 1*). The study team then organized these strategies by two adaptation goals, which were defined by the investigative team in collaboration with clinic and community partners during the project development phase. Strategies focused on *improving the effectiveness of result communication* were aligned and selected for subsequent implementation (*n* = 2), while strategies aimed to *increase the number of women screened* fell into two workflow categories: appointment scheduling (*n* = 9) and follow-up processes (*n* = 9). As implementing all 18 strategies was not feasible or sustainable, they were prioritized through all-stakeholder deliberations that engaged both community members and internal stakeholders.

Stakeholders (*N* = 12) rated each strategy based on four criteria: acceptability, feasibility, effectiveness, and sustainability. During the virtual deliberation session, participants received definitions of the rating criteria before rating the strategies. As shown in Table 1, strategies fell into three distinct rating categories. The high-scoring strategies (*n* = 7) received consistently high scores from both community and stakeholder groups. These included promoting screening via social media and community outreach, using clinic spaces for awareness, and expanding screening into non-women’s health programs. In contrast, the mixed-scoring strategies (*n* = 7) were rated higher by community members than by stakeholders. These strategies often involved proactive patient outreach (e.g., phone calls, text messaging), options for patients to complete screening outside clinic visits, and automated clinic EMR follow-up systems. Group deliberation revealed that while these strategies reduce patient burden and promote access, stakeholders had concerns about staff time, IT capacity, and policy compliance. Additionally, online self-completion was viewed as nonviable due to concerns about low health literacy and the need for provider-facilitated result communication, as well as the ability to ensure timely referral and follow-up. Finally, the low-scoring strategies (*n* = 4), such as mobile clinics, paper screeners, and transportation vouchers, received consistently low ratings (mean scores < 14) from both groups due to concerns about resource intensity and long-term sustainability.

**Table 1:** Community member and stakeholder ratings of proposed adaptation strategies to increase family cancer history screening uptake among women in rural Georgia, on acceptability, feasibility, effectiveness, and sustainability, United States, 2024

Categories	List of Strategies	Mean Scores for Acceptability, Feasibility, Effectiveness, and Sustainability		
		All Stakeholders (*N* = 12)	Community Members (*n* = 6)	Stakeholders (*n* = 6)
High-scoring	Clinic staff should briefly discuss the importance of follow-up for high-risk family cancer history screening results with patients.	17.59	18.17	17.00
	The Department of Public Health (DPH) should promote family cancer history screening by sending staff to health fairs, having a presence at local events, and using social media promotion.	16.83	17.33	15.50
	Clinic staff should raise awareness of family cancer history screening by setting up a table in the lobby, putting up posters in waiting rooms, and distributing flyers for patients to read while they wait.	16.50	16.17	16.83
	The DPH’s IT department should build the screening tool into the electronic medical record (EMR) system allowing nurses who provide other services (e.g., STD screening, immunizations, and TB treatment) to screen patients.	16.33	15.83	16.83
	The DPH should highlight the family cancer history screening program at the Health Promotion and Community Resource Consortium to raise awareness among other agencies.	15.58	16.50	14.67
	Clinic staff should send follow-up letters to patients’ homes to remind them of actions needed for high-risk screening results.	15.50	14.67	16.33
	The DPH’s IT department should add reminder banners to the EMR system to prompt nurses to screen patients.	15.25	15.67	14.83
Mixed-scoring	Clinic staff should have a standardized protocol that requires them to mention family cancer history screening to patients who call about other services.	16.17	19.17	13.00
	The clinic should use the EMR system to send text messages to patients about screening.	14.92	17.17	12.83
	Clinic staff should offer patients the option to complete family cancer history screening over the phone.	15.67	17.67	13.67
	The DPH should allow patients to complete the screening on Georgia CORE’s website, either in the waiting room or at home, with results automatically integrated into the EMR system.	14.75	16	13.5
	The DPH’s IT department should implement referrals, result sharing, and follow-ups into the EMR system, using features like a tickler system for PAP smear results.	14.75	16.33	13.17
	Clinic staff should call patients to confirm they were contacted by Georgia CORE about high-risk screening results and help with any needed connections.	14.42	15.17	13.67
	The DPH’s IT department should develop a system that allows the EMR to automatically pull and transfer family history information to Georgia CORE’s online screener.	14.25	15.33	13.17
Low-scoring	The DPH should provide family cancer history screening through mobile health clinics in the community.	13.50	14.33	13.00
	Georgia CORE should call patients with high-risk screening results using a recognizable 404 area code.	13.50	13.83	13.17
	The clinic should provide a paper screener for patients to fill out while waiting, and then nurses can input the answers into the computer.	13.42	12.00	14.83
	The clinic should provide transportation vouchers or other means to help patients attend appointments.	13.17	14.00	12.33
	*Scores were ranked based on mean sum scores across all stakeholders.*			

*Abbreviations*: *DPH* Department of Public Health, *EMR* Electronic medical record, *IT* Information technology, *STD* Sexually transmitted disease, *TB* Tuberculosis, *Georgia CORE* Georgia Center for Oncology Research and Education

### Final adaptation strategies for screening program implementation

We finalized 15 prioritized adaptation strategies corresponding to four key procedures in the screening workflow (Fig. 3).

Scheduling the screening: No existing scheduling components were removed. New elements are added to enhance outreach and engagement, including social media posts, in-person events, and in-clinic promotional materials (e.g., posters, stands). An automated phone greeting and a standardized one-sentence script for front desk staff will be implemented to promote screening during patient calls. The screening program is expanded to all health programs within the clinic, except for child health. 

Administering the screening: The FHAT remains unchanged. To support delivery, county-specific screening web links replace the dropdown menu to reduce data entry errors. Screening banners are incorporated into the clinic workflow to prompt providers to assess eligible patients. The adapted Georgia CORE screening website (version 2.0) removes redundant steps (e.g., collect patient data use agreements) and clarifies field labels (e.g., “zip code” to “patient zip code”). 

Discussing screening results: The in-person interaction remains unchanged. After screening, the clinic provider offers a brief verbal explanation of the results and emphasizes the importance of follow-up when applicable. Risk-stratified result summaries and resource pages are provided for both positive and negative results to reduce provider burden and support patient understanding. The adapted Georgia CORE website includes a consent checkbox to enable Georgia CORE follow-up communications. For patients who screen positive, Georgia CORE sends automated follow-up messages via text and email immediately after the clinic visit, and again at 3 days, 7 days, 2 weeks, 6 months, and 1 year to encourage uptake of genetic counseling and access to resources related to increased cancer risk. Patients who screen negative also receive automated follow-up messages, including their screening results and recommendations to undertake routine mammograms starting at age 40, regularly update family cancer history, and follow evidence-based healthy lifestyle guidelines. All patients receive their result summary printed on a wallet card the size of a credit card for easy storage. 

Follow-ups: A simplified result documentation system of the patient risk assessment is implemented in the clinic EMR system, recording only positive or negative status, and thus replacing the earlier, more detailed result summaries. The manual referral process (e.g., print and fax) is removed and replaced with an automated referral system integrated into Georgia CORE’s website version 2.0. Providers can obtain consent and submit referrals directly through the platform by clicking a single referral button. The updated system also consolidates follow-up tracking for both positive and negative results into one interface. 

All 15 adaptations were planned, proactive, and fidelity-consistent (Tables 2 and 3). Most aimed to improve the number of women screened (*n* = 13, 87%), while a smaller number focused on enhancing communication of screening results (*n* = 2, 13%). The majority of adaptations (*n* = 10, 67%) targeted implementation and program expansion activities, followed by changes to context (*n* = 4, 26%) and content (*n* = 1, 7%). Approximately half of the adaptations were made at the clinic level (*n* = 7, 47%), with additional adaptations at the organizational (Georgia CORE) (*n* = 3, 20%), individual (*n* = 2, 13%), community (*n* = 2, 13%), and provider (*n* = 1, 7%) levels. Most adaptations (*n* = 10, 67%) involved adding new components, while others involved expanding (*n* = 1, 7%), reducing (*n* = 2, 13%), removing (*n* = 1, 7%), and maintaining (*n* = 1, 7%) existing processes.

**Table 2:** Adaptation strategies selected for implementation of a family cancer history screening program, by screening workflow step, level of delivery, and nature of modification, Georgia, United States, 2025

Adaptations Made		What	Level of Delivery	Nature of Modification
Adaptation Goal 1: Increase the number of women screened				
Step 1	The DPH promotes screening at community events and on social media.	Implementation and Scale-up Activities	Community	Add
	Clinic staff sets up stands, posters, and digital signs in the clinic.	Implementation and Scale-up Activities	Clinic	Add
	Clinics add an automated phone greeting to promote the screening program.	Implementation and Scale-up Activities	Clinic	Add
	Clinic staff uses a one-sentence phone script to promote screening.	Implementation and Scale-up Activities	Provider	Add
	The DPH promotes the program through community consortium partnerships.	Implementation and Scale-up Activities	Community	Add
	Clinics expand the screening to other health programs.	Contextual	Clinic	Expand
	Clinics embed screening reminder banners into the EMR workflow.	Implementation and Scale-up Activities	Clinic	Add
Step 2	Clinics use a county-specific screening link to streamline data collection.	Contextual	Clinic	Add
	Georgia CORE adapted screening website removes unnecessary data collection.	Contextual	Organization	Remove
	Clinics maintain in-person interaction.	Contextual	Clinic	Maintain
Step 3	Clinics simplify the result documentation process.	Implementation and Scale-up Activities	Clinic	Reduce
Step 4	Georgia CORE adapted screening website adds an automatic referral system for positive screens.	Implementation and Scale-up Activities	Organization	Add
	Georgia CORE adapted screening website consolidates case tracking systems into one.	Implementation and Scale-up Activities	Organization	Reduce
Adaptation Goal 2: Improve screening results communication				
Step 3	Georgia CORE adapted screening website adds theory-based communication materials to facilitate screening result comprehension for both positive and negative results.	Content	Individual	Add
Step 4	Georgia CORE adapted screening website adds automatic email/text message follow-ups to promote risk-stratified cancer screening behaviors for both positive and negative results.	Contextual	Individual	Add

Nature of modification categories indicate whether an existing program element was added, expanded, maintained, reduced, or removed during the adaptation process

*Abbreviations*: *DPH* Department of Public Health, *EMR* Electronic medical record, *Georgia CORE* Georgia Center for Oncology Research and Education

**Table 3:** Summary of adaptations to a family cancer history screening program in Georgia, United States, characterized using the Framework for Reporting Adaptations and Modifications-Enhanced (FRAME)

FRAME codes	*n* (%)
Modification Timing	
When did the modification occur?	
Pre-implementation phase only	15 (100%)
Whether the modification was planned?	
Planned/proactive	15 (100%)
Modification Process	
Why was the modification made?	
Improve the number of women screened	13 (87%)
Improve screening results communication	2 (13%)
Was the modification fidelity-consistent?	
Consistent	15 (100%)
What was modified?	
Context	4 (26%)
Content	1 (7%)
Implementation and scale-up activities	10 (67%)
At what level of delivery was the modification made?	
Individual (patient)	
Provider	2 (13%)1 (7%)
Clinic	7 (47%)
Organization	3 (20%)
Community	2 (13%)
What was the nature of the modification of contents?	
Add	10 (67%)
Expand	1 (7%)
Remove	1 (7%)
Reduce	2 (13%)
Maintain	1 (7%)

*Abbreviation*: *FRAME* Framework for Reporting Adaptations and Modifications–Enhanced

## Discussion

We are among the first to apply an implementation science framework to systematically document pre-implementation adaptations to a population-based family cancer history screening program in a rural public health setting. Conducted in Southwest Georgia, a region with historically low engagement in genetic screening, our findings show how processes partnering constituent groups can guide the adaptation of evidence-based interventions to fit local healthcare settings. By applying the FRAME, we documented proactive, fidelity-consistent modifications and contributed a case example to the growing literature on population-based genetic screening implementation.

The inclusion of stakeholders across organizational, provider, community, and patient levels was critical for identifying where adaptations would be most advantageous and feasible. Evidence consistently shows that diverse stakeholder engagement leads to better outcomes for vulnerable populations and enhances the acceptability and effectiveness of interventions [27–29]. Notably, community members prioritized strategies to reduce patient burden and improve access, while clinic staff emphasized practical concerns such as the availability of adequate staffing, IT infrastructure, and DPH patient communication policy compliance. We acknowledge that not all 14 clinics in the district were represented across every adaptation step, which may have influenced the relative prioritization of adaptations. However, some of those interviewed work in multiple clinics and therefore may offer perspectives beyond the 6 participating clinics. Nevertheless, the structured deliberation process enabled participants to interpret ratings by weighing trade-offs and community-level impacts, yielding in-depth insight into community priorities despite differing viewpoints.

Another key insight from our adaptation process is the importance of parsimony when reconciling differing priorities and guiding decision-making. While multilevel approaches are often promoted to address health disparities, resource constraints frequently limit their feasibility in public health settings. Consistent with the recommendation from McBride et al. [30] to prioritize levels of influence based on contextual fit, most of our adaptations focused on clinic-level program activities, such as workflow integration and EMR prompts that could impact all patients, that have wider impact. These changes aligned with current operations and required minimal infrastructure or personnel investment. In contrast, few adaptations were made at the provider or patient level, where resource-intensive training or tailoring was less feasible. These findings highlight that not all levels of implementation carry equal weight across settings. Focusing on priority factors identified by stakeholders may improve effectiveness and increase the likelihood of sustainability.

The adaptation process also highlights the importance of anticipating unintended consequences when selecting and implementing strategies. For example, while having patients self-complete screening online could theoretically increase access, concerns about digital literacy, the need for provider-facilitated communication, and gaps in medical record integration made such online screening unsuitable for the current setting. This finding is consistent with broader evidence that adaptations, if not tailored to the local setting, can inadvertently undermine equity or compromise the intervention’s core functions [30, 31].

Our adaptation approach may inform future efforts to improve uptake and sustainability of other population-based health programs in public health settings, particularly in underserved regions. To ensure the consideration of multilevel factors that influence program outcomes, we used the EPIS framework to structure the adaptation process by key stages of pre-implementation: Exploration (assessing needs, context, and fit across system, organization, and community levels) and Preparation (engaging stakeholders to develop adaptation components and implementation strategies) prior to our trial implementation. The FRAME framework further supported systematic documentation and categorization of the adaptations we made using its domains, including what was modified, at what level, whether changes were planned, and the nature and rationale of each modification, thus enhancing transparent decision-making and adaptation in other settings. Multiple constituent engagement using deliberation strategies facilitated in-depth discussion and careful weighing of program priorities. Additionally, anticipating unintended consequences of adaptation strategies, particularly for subgroups that may face greater barriers, emerged as a necessary step to avoid adaptations that could inadvertently exacerbate inequities. Our emphasis on parsimony helped prioritize intervention levels and strategies that were more feasible and likely to be effective within existing resources.

This study has several limitations. Our approach was resource- and time-intensive, which may limit feasibility in settings with fewer supports. In addition, the study was conducted in a single, though large, public health district spanning 14 counties. While several identified barriers and facilitators are likely relevant to similar public health systems, findings should be interpreted with caution regarding broader generalizability. Finally, the current family cancer history screening program is delivered through the Women’s Health Section of participating clinics and is therefore limited to women, and it focuses on hereditary risk for *BRCA*-associated cancers rather than the larger population with a cancer family history that may warrant earlier breast or colorectal cancer screening. Building on our adapted approach, future implementation research could investigate extending hereditary cancer screening to additional clinical programs to reach broader patient populations and incorporate referrals for other cancer screenings.

## Conclusions

Strong partnerships among public health agencies, community organizations, and academic institutions were essential for guiding the adaptation process in this study. Applying implementation science frameworks to design and document pre-implementation adaptations, combined with multilevel stakeholder engagement and explicit attention to sustainability, resulted in a systematic and transparent approach to program adaptation. This stepwise process may inform future efforts to scale and sustain population-based hereditary cancer screening programs in underserved public health settings.

## Supplementary Information

Supplementary Material 1.

Supplementary Material 2.

## Data Availability

The datasets used and/or analyzed during the current study are available from the corresponding author on reasonable request.

## Abbreviations

ACGN: Advanced Clinical Genomics Nurse
BCCP: Breast and Cervical Cancer Program
BRCA: BReast CAncer gene
CDC: Centers for Disease Control and Prevention
CFIR: Consolidated Framework for Implementation Research
Georgia CORE: Georgia Center for Oncology Research and Education
DPH: Department of Public Health
EMR: Electronic Medical Record
EPIS: Exploration, Preparation, Implementation, and Sustainment
FHAT: Family History Assessment Tool
FRAME: Framework for Reporting Adaptations and Modifications-Enhanced
IT: Information Technology
RE-AIM: Reach, Effectiveness, Adoption, Implementation, and Maintenance
USPSTF: U.S. Preventive Services Task Force
