# Supplementary material for: A multilevel adaptation approach for the sustainable scale-up of family cancer history screening in rural public health clinics
Source: Arch Public Health. 2026 Jul 17;84:221. doi: 10.1186/s13690-026-02018-7 (PMC13660225; doi:10.1186/s13690-026-02018-7)
Supplement: Supplementary file 1 [file 13690_2026_2018_MOESM2_ESM.pdf]

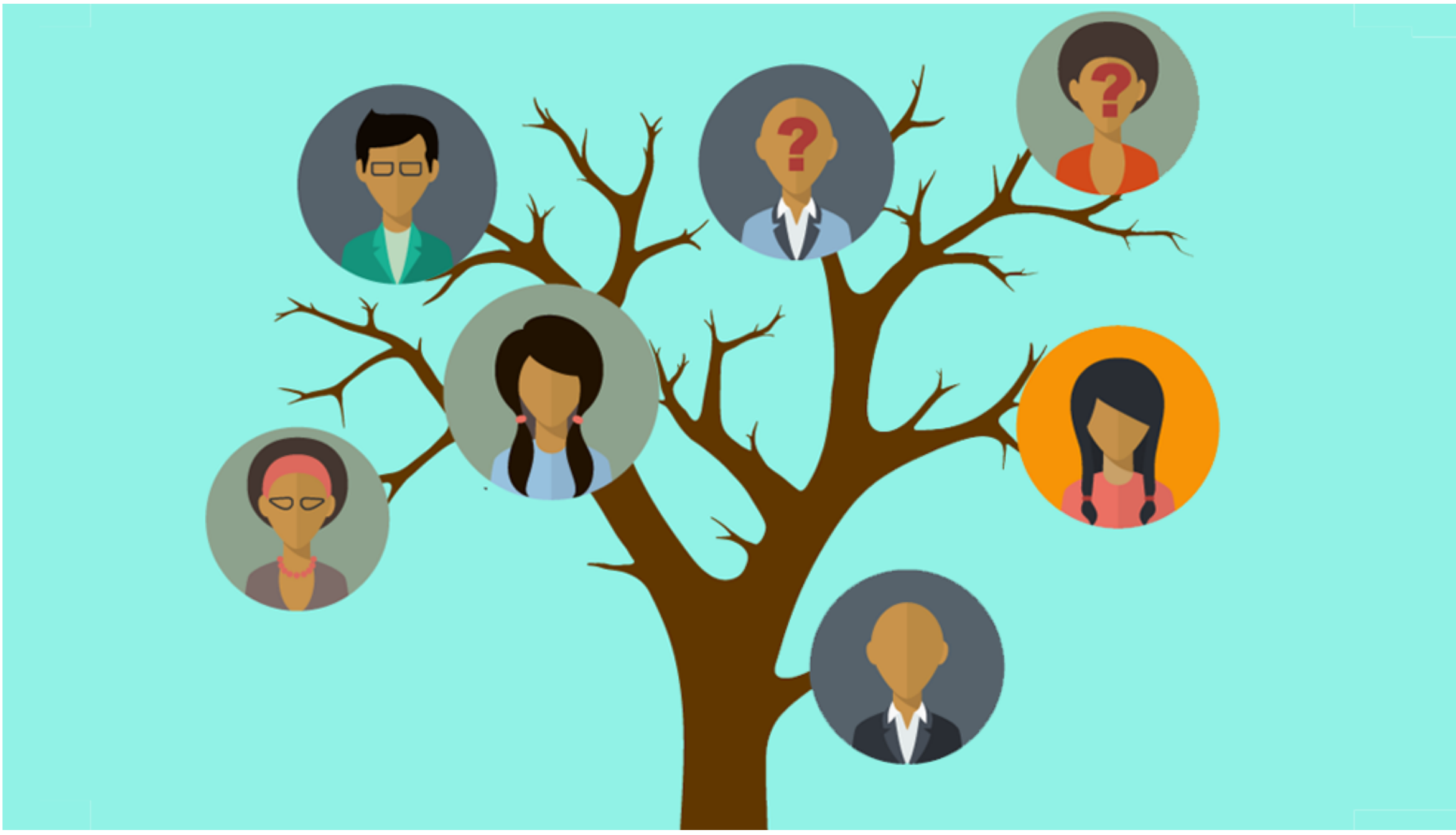

STAKEHOLDER MEETING

ON

INCREASING WOMEN'S ACCESS TO INHERITED CANCER RISK SCREENING

Participant Workbook

Tuesday, June 18th 2024

8:00 AM - 9:30 AM

EMORY UNIVERSITY

ROLLINS SCHOOL OF PUBLIC HEALTH

Questions? Email the Project Manager, **Shacoria Anderson**, at shacoria.anderson@emory.edu

Zoom Link: https://rb.gy/8erxnp

Passcode: 01234

Table of Contents

Introduction to the Project 3

Study Goals..........4

Meeting Participants 5

Pre-session Materials..........6

Session Agenda..........8

Guiding Principles for the Stakeholder Discussion Group..........9

Background – Georgia CORE Inherited Cancer Risk Screener..........10

Inherited Cancer Risk Screening Clinic Workflow Diagram..........11

Small Group Session1: Gaps and Opportunities in Scheduling Inherited Cancer Risk Screening..........12

Small Group Session 2: Gaps and Opportunities in Completing Inherited Cancer Risk Screening and Patient Follow-Ups 13

Appendix: Complete Gaps/Opportunities Table 14

Introduction to the Project

Welcome! You are here today because you live in the Southwest Public Health District, and you were interested in sharing your opinion about the public health clinic's approach to inherited cancer risk screening.

Georgia is the only state that has a population-based screening program for hereditary breast and ovarian cancer. The Georgia Breast Cancer Genomics Project started in 2012. It is a voluntary program, in which family history screening is offered in women's health section at public health clinics throughout Georgia.

During this **90-minute Zoom meeting**, you will learn about the current process of the inherited cancer risk screening in public health clinics. We will present the gaps and opportunities for increasing the number of women screened. Afterward, we will ask for your thoughts and opinions on the strategies presented. Our goal is to use your feedback to make changes to the screening program.

This is an open discussion where you are able to share your opinions with others in a respectful way. **There are no right or wrong answers, but rather different points of view**.

The event begins at 8:00 am on Tuesday, June 18th, 2024. Please plan to log on **5 minutes early** to register and to gather and organize any materials you may need for the sessions.

Study Goals

To discuss and evaluate strategies to:

1. **Increase the number of women screened by Georgia's Inherited Family History Screening Program.**
2. **Ensure that these efforts are sustainable in public health clinics.**

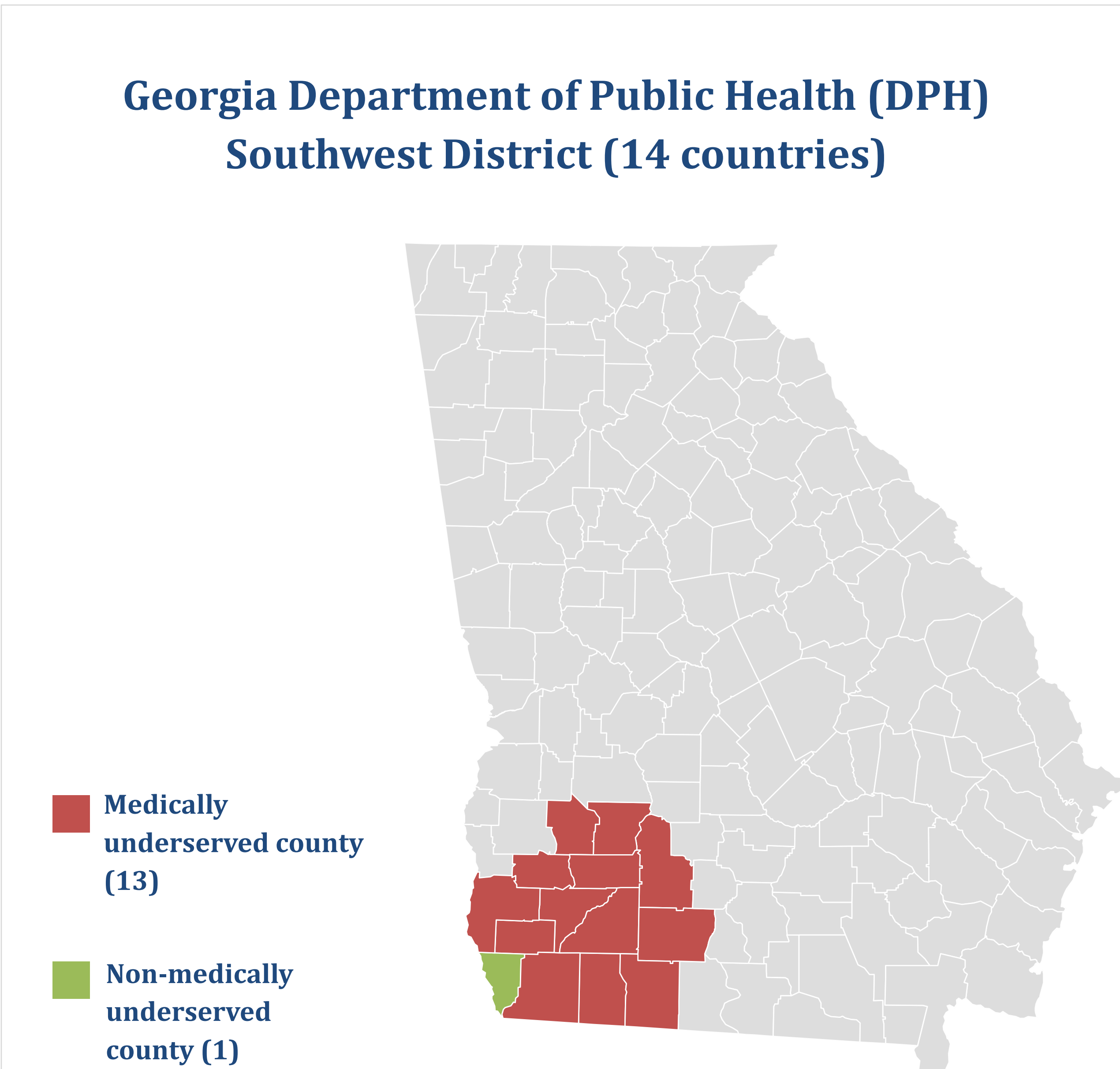

Meeting Participants

To learn more about our study team, please refer to the table below.

Name	Affiliation
**Sheryl Gabram**	Georgia Center of Oncology Research and Education
**Cindy Snyder**	Georgia Center of Oncology Research and Education
**Cynthia Walters**	Georgia Department of Public Health Southwest District
**Michele Gornick**	Emory University
**Denise Ballard**	Emory University
**Dayanna Ramirez**	Emory University
**Angela Zhang**	Emory University
**Shacoria Anderson**	Emory University
**Colleen McBride**	Emory University
**Yue Guan ("Guan")**	Emory University

To learn more about our community representatives, please refer to the table below.

Name	Affiliation
**Lauren Mitchell**	RN, Worth County
**Laura Bosely**	Customer Service Rep, Thomas County
**Kimberly Sherrer**	Billing Clerk Supervisor
**Carlyle Giannars**	Family Nurse Practitioner
**Sunshine Rowles**	Family Nurse Practitioner
**Theresa Weiss**	Hospitalist Research Nurse-Phoebe Health
**Lasaunda Garrett**	Lead Patient Navigator, Albany Area Primary Health Care
**Ashley Heredeen**	Screening Program Manager, Horizons
**Lucinda Lakin**	Southwest Georgia Community Member
**Shaquille Minter**	Southwest Georgia Community Member
**Kerrie Davis**	Southwest Georgia Community Member
**Harriet Hollis**	Southwest Georgia Community Member
**Kay Eady**	Southwest Georgia Community Member
**La'Quevia Sheffield**	Southwest Georgia Community Member
**Madhu Kuntawala**	Southwest Georgia Community Member

Pre-Session Materials

The study team interviewed 11 internal stakeholders in the Southwest Public Health District, including healthcare providers and clerical staff, to discuss their experiences with the inherited cancer risk screening program. They also identified gaps and opportunities in the process. On the day of our meeting, you will have time to review and select your top choices from the strategies listed below, which are related to two key procedures: scheduling family history screening (session 1) and completing family history screening and follow-ups (session 2).

Session 1: Scheduling Family History Screening

Thinking about **how easy, doable, effective, and long-lasting** these strategies are, which three do you think would most increase the number of women screened if public health clinics used them? **Please pick your top 3 choices.**

Gaps	Strategies
• The women who are not being screened include those who visit the public health clinic for other services and those who face transportation barriers.	1.1 The Department of Public Health (DPH) should promote family history screening by sending staff to health fairs, having a presence at local events, and using social media promotion.
	1.2 The DPH's IT department should build the screening tool into the electronic medical record (EMR) system allowing nurses who provide other services (e.g., STD screening, immunizations, and TB treatment) to screen patients.
	1.3 Clinic staff should raise awareness of family history screening by setting up a table in the lobby, putting up posters in waiting rooms, and distributing flyers for patients to read while they wait.
	1.4 Clinic staff should have a standardized protocol that requires them to mention the family history screening to patients who call about other services.
	1.5 The clinic should use the EMR system to send text messages to patients about screening.
	1.6 Clinic staff should offer patients the option to complete family history screening over the phone.
	1.7 The clinic should provide transportation vouchers or other means to help patients attend appointments.
	1.8 The DPH should highlight the family history screening program at the Health Promotion and Community Resource Consortium to raise awareness among other agencies.
	1.9 The DPH should provide family history screening through mobile health clinics in the community.

Pre-Session Materials

Session 2: Completing Family History Screening and Follow Ups

Thinking about **how easy, doable, effective, and long-lasting** these strategies are, which three do you think would most increase the number of women screened and followed up with if public health clinics used them? **Please pick your top 3 choices.**

Gaps

- Some workflow steps are repeated, like asking patients for the same family history information multiple times during a visit.
- Some patients are not followed up with after screening because they don't recognize Georgia CORE's area code when called, referrals are sent by fax, and there is no follow-up tracking system.

Strategies

2.1 The DPH should allow patients to complete the screening on Georgia CORE's website, either in the waiting room or at home, with results automatically integrated into the electronic medical record (EMR) system.

2.2 The clinic should provide a paper screener for patients to fill out while waiting, and then nurses can input the answers into the computer.

2.3 The DPH's IT department should develop a system that allows the EMR to automatically pull and transfer family history information to Georgia CORE's online screener.

2.4 The DPH's IT department should implement referrals, result sharing, and follow-ups into the EMR system, using features like a tickler system for PAP smear results.

2.5 The DPH's IT department should add reminder banners to the EMR system to prompt nurses to screen patients.

2.6 Clinic staff should briefly discuss the importance of follow-up for high-risk family history screening results with patients.

2.7 Clinic staff should call patients to confirm they were contacted by Georgia CORE about high-risk screening results and help with any needed connections.

2.8 Clinic staff should send follow-up letters to patients' homes to remind them of actions needed for high-risk screening results.

2.9 Georgia CORE should call patients with high-risk screening results using a recognizable 404 area code.

Meeting Agenda

Time	Duration	Activity & Topic
8:00am-8:05am	5 min	**Welcome**<br>*Stakeholder Meeting on Increasing Women's Access to Inherited Cancer Risk Screening*<br>● Introduction to the study, team, and community groups<br>● Review session agenda and group exercises
**Session 1: Scheduling Inherited Cancer Risk Screening**		
8:05am-8:10am	5 min	**Full Group Session:**<br>● Review identified gaps and opportunities in inherited cancer risk screening
8:10am-8:35am	25 min	**Small Groups:**<br>● Complete survey<br>● Discuss prioritized strategies
8:35am-8:45am	10 min	**Full Group Session:**<br>● Report small group discussion to the full group
**Session 2: Completing Inherited Cancer Risk Screening and Patient Follow-Ups**		
8:45am-8:50am	5 min	**Full Group Session:**<br>● Review identified gaps and opportunities in inherited cancer risk completion and follow-ups
8:50am-9:15am	25 min	**Small Groups:**<br>● Complete survey<br>● Discuss prioritized strategies
9:15-9:25am	10 min	**Full Group Session:**<br>● Report small group discussion to the full group
9:25am-9:30am	5 min	**Wrap-up, review, and thank you**

Guiding Principles for the Stakeholder Discussion Group

Listen with care.
Speak with each other while remembering each person has thoughts, ideas, feelings, and experiences that shape their story. Look for common goals and interests. Be open to different points of view. Listen carefully to the ideas of others. Don't interrupt each other.

Maintain focus.
Focus on issues, not people. Express real concerns as they relate to your own thoughts. Understand and focus on the topics. Limit side topics, and avoid side conversations. Stay focused; minimize stories unless they are really important to the issues.

Maintain a positive attitude.
Assume good motives; do not look for hidden agendas. If the meaning of something said was not clear, ask questions and seek to understand. Be thoughtful about the diversity in communication styles - some people speak directly and some are storytellers.

Practice brevity and clarity.
Speak clearly and concisely. Unless asked, limit discussion of an issue or item to as short a statement as possible. Try to stay on the theme of discussion. Allow the facilitator to keep the discussion moving. Respect the timelines of the agenda.

Participate fully.
Participate and speak up during discussions, but do not dominate through interruptions or long-windedness. Express views rather than remaining silent during any disagreements.

Disagree positively.
Respect the right to disagree-respectfully. Be a problem solver by suggesting better approaches or solutions. Adopt a cooperative attitude; look for opportunities to make changes that work for all parties.

Georgia’s Inherited Cancer Risk Screening Tool

About This Tool

Georgia CORE’s Breast & Ovarian Cancer Genetics Referral Screening Tool is an online tool that asks about your family cancer history to assess for hereditary cancer risks. It helps quickly identify who should be referred for cancer genetic counseling and might need genetic testing.

Nurses in Public Health Clinics help women complete this tool during their visit.

You can also complete the screener in English and Spanish on the Georgia CORE website:

https://www.georgiacancerinfo.org/breast-cancer-gene-screen

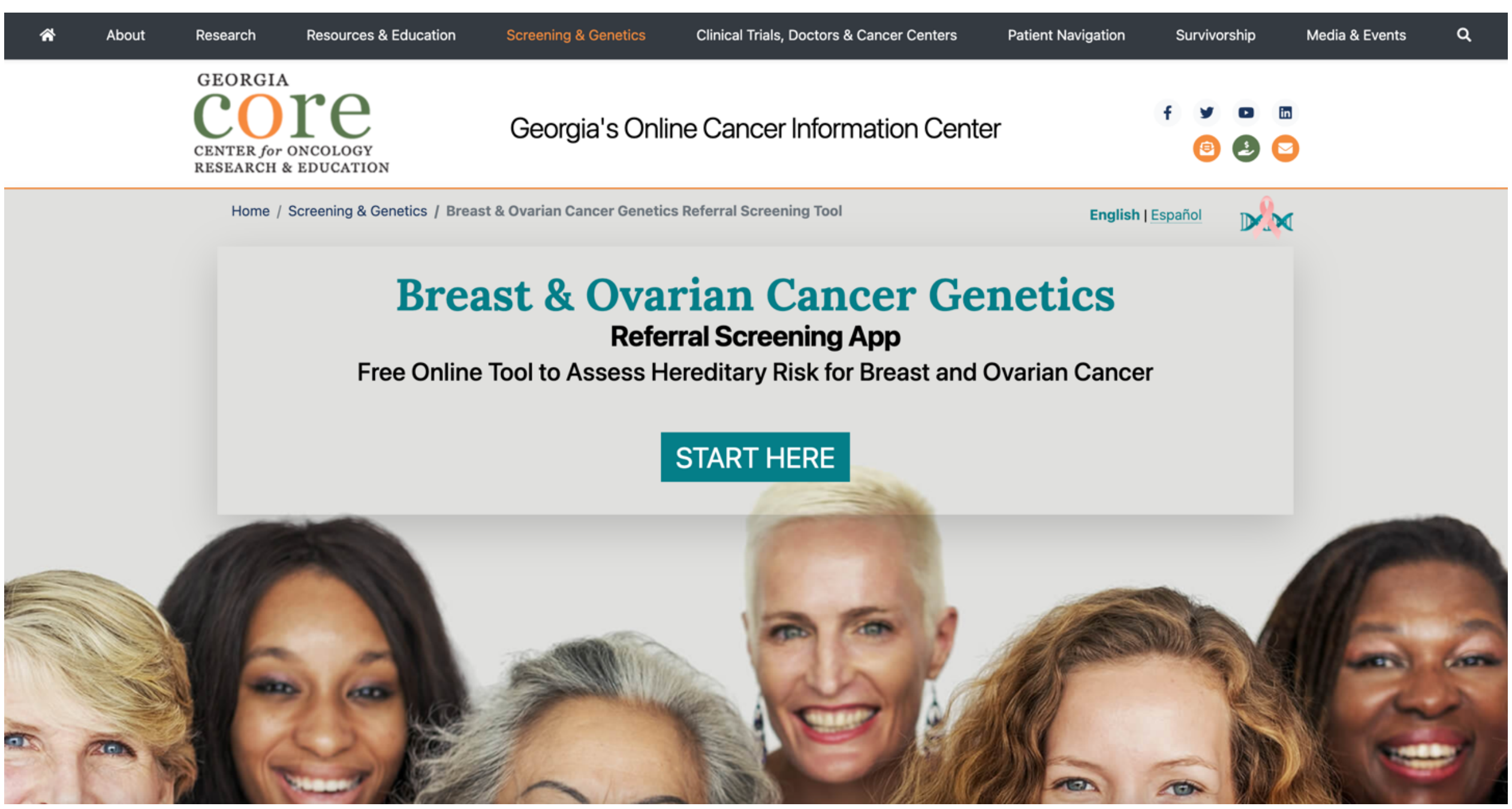

Inherited Cancer Risk Screening Workflow Diagram

About

Step 1

A clerical staff member or nurse will receive the call to schedule an appointment or agree to a walk-in.

Step 2

A woman will check-in and submit her vitals. The nurse will then introduce the topic of inherited cancer, family history screening, and the complete the form on the Georgia CORE website with the patient in a private exam room.

Step 3

The nurse will discuss the result of the family history screener with the patient and update the electronic health record.

Step 4

If the patient receives a ***positive result***, the nurse will complete the Georgia CORE referral form, update the patient chart, and fax the results to Georgia CORE. If the patient received a ***negative result***, the nurse would continue filling out the family history screener at the patient's annual healthcare visit to keep the patient's family health history, and relative risk, updated in the system.

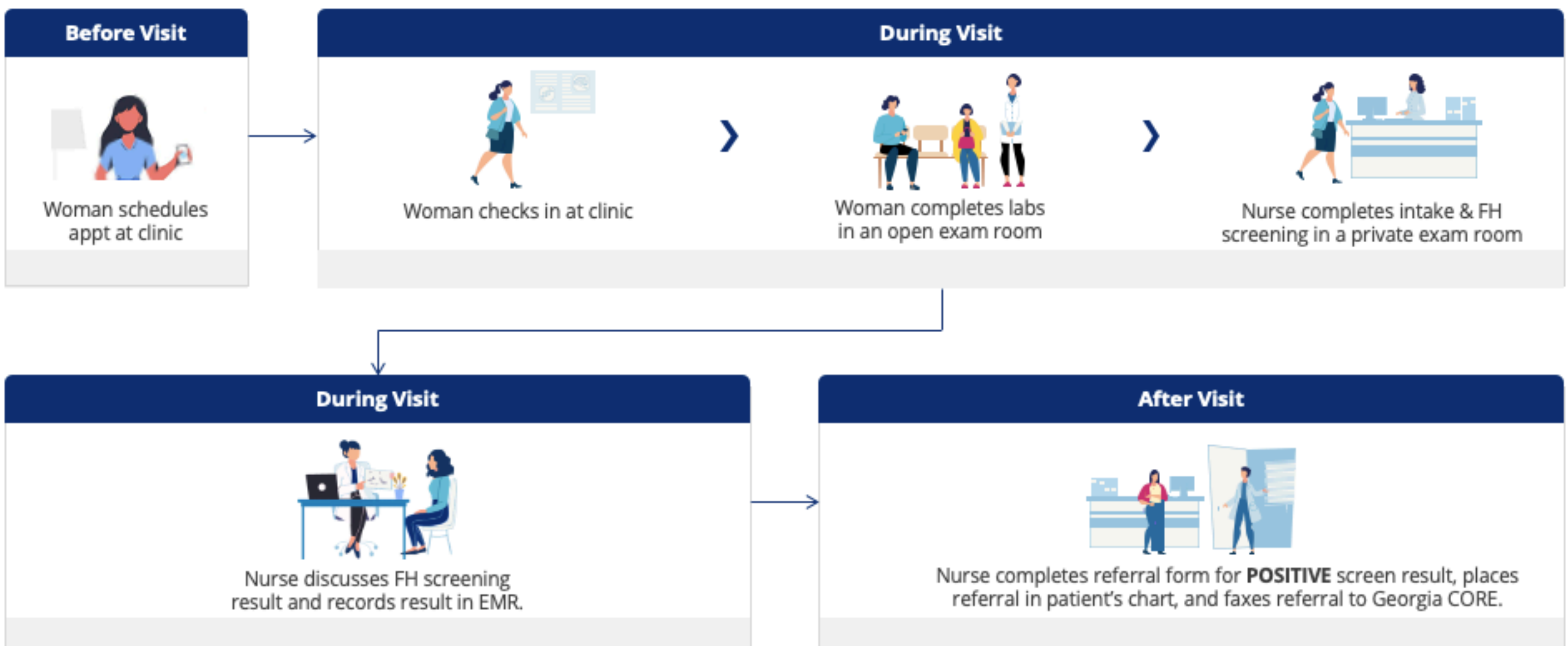

<u>Small Group Session 1:</u> Gaps and Opportunities in Scheduling Inherited Cancer Risk Screening

Internal stakeholders, like healthcare providers and clerical staff, identified gaps and opportunities related to scheduling family history screening. You can find the full table of these gaps and opportunities in the Appendix on Page 11.

NOTES:

Small Group Session 2: Gaps and Opportunities in Completing Inherited Cancer Risk Screening and Patient Follow-Ups

Internal stakeholders, like healthcare providers and clerical staff, identified gaps and opportunities related to scheduling family history screening. You can find the full table of these gaps and opportunities in the Appendix on Page 11.

NOTES:

Appendix

The study team interviewed 11 internal stakeholders, like healthcare providers and clerical staff, to discuss their experiences with this inherited cancer risk screening program. Each participant identified gaps and opportunities related to 1) scheduling inherited cancer risk screening appointments, 2) completing inherited cancer risk screening, 3) discussing screening results with patients, and 4) completing patient follow-up. During the meeting, we will not discuss gaps and opportunities related to result communication, as these will be addressed in a subsequent stakeholder working group. However, we would like to share the full list with you for your reference.

	Gaps Mentioned	Opportunities to Fill Gaps Mentioned
**Step 1: Scheduling appointment at clinic.**	1. Missing women for screening who visit the clinic for other programs/services, including women who visit for STD screening, immunizations, and TB patients (n=6).	• Incorporate screening into other appointments within clinic (for example, nurses who provide immunizations can give brochure about screening to patients or refer eligible patients for screening) (n=6)<br>• Increase community outreach to promote screening (i.e., attend health fairs and local events such as walks) (n=4)<br>• Set up table in clinic lobby to educate patients who come into clinic about the screening program (n=2)<br>• Train nurses in other clinic programs that may be less familiar with the screening program so they are educated about screening (n=2)<br>• Use flyers within clinic to promote screening (n=1)<br>• Mention screening to patients who call about other services (n=1)<br>• Including screening in EMR system across departments and allowing patients to opt-in from other programs in the clinic (n=1)<br>• Hire a breast and cervical cancer program navigator (n=1)<br>• Social media promotion (n=1)<br>• Representation of breast and cervical cancer program at health promotion and community resource consortium to make more programs aware of screening (n=1).
	2. Missing women who are uninsured, Spanish-speaking, or have transportation barriers (n=4).	• Implement more telehealth services and complete screening over the phone (n=2)<br>• Offer mobile health clinic to complete screening within the community (n=2)<br>• Offer patients the ability to complete screening online (n=1)<br>• Have someone attend community events and increase awareness about screening (n=1)

	Gaps Mentioned	Opportunities to Fill Gaps Mentioned
		• Arrange transportation to clinic (n=1).
	3. Limited staff availability makes it difficult to accommodate all patients (n=2).	None mentioned.
	4. Some women may miss their appointments (n=1).	• Appointment reminders: calling patients the day of their appointment to remind them seems to be most effective (n=1).
	5. Could make improvements with how walk-ins are handled, as some clinics may be able to provide the service to patients and others may have to reschedule them to come in at another time (n=1).	None mentioned.
**Step 2: Check-in, intake, and FH completion.**	1. Repetitive workflow steps (similar information about family history being asked multiple times during visit). (n=7)	• Have the patient complete screening on the website on their own and integrate with VHN/EMR system to automatically pull patient's results into their chart (n=2)<br>• Incorporating results from medical history and family history and pulling in these results into the VHN (n=2)<br>• Can transfer answers from medical history to the Georgia CORE website questionnaire (n=2)<br>• Having the questions on a paper screener for patients to fill out while they wait to be seen and the nurse can input answers into computer (n=2).<br>• Knowing which questions are repetitive so you don't ask more than once and fill in the information in both sections while the patient is talking (n=1).<br>• Merge some of the questions together (n=1).
	2. Limiting the completion of the screening form to just clinical	• Similar to the diabetes prevention program, could conduct screening at health fairs or community events to assess risk and refer patients to next steps (n=1)

	Gaps Mentioned	Opportunities to Fill Gaps Mentioned
	staff may limit the accessibility of the form to others. (n=1)	• Other clinic staff from other programs could complete an initial assessment of patient risk (n=1). To complete this, would need staff who are trained to use the assessment and be able to refer patient to next steps.
	3. Some patients may seem that they're in a hurry and may not be as engaged at first (n=1)	• Once the nurse fully educates the patient on the importance of the screening and knowing their risk, the patient is likely to take it more seriously (n=1).
	4. Repetitiveness with check-in process, specifically the completion of written consent forms. (n=1)	• Clinic transitioning from paper to electronic process may help reduce this repetitiveness (n=1).
**Step 3: Discussion of screening results.**	1. High turnover of nurses and providers (n=1)	• Making sure the nurses are well-trained on how to discuss results with patients and providing education to patients; maybe incorporating a training for providers that was more interactive or providing something they can keep in their notebooks and refer back to (n=1)
	2. Forgetting to record screening results on EMR (n=1)	• Automatically populate results in the EMR system instead of having to input them manually (n=1)
	3. Using language line is time consuming (n=4)	• If you anticipate a patient may have a language barrier, try to schedule more time into their appointment to accommodate the time needed to use the language line (n=1).
	4. Language barriers between providers and patients (n=7); patients may call back to clarify information they received during their visit	• Use language lines (n=4); make sure clinics know this is a resource available to them.<br>• Have educational materials available in Spanish (n=3).<br>• Increased clinic access to interpreters (n=2).<br>• Make sure an interpreter is available during the patient's appointment (n=1).
	5. Limited time during clinic appointments (n=3); limits opportunities for patients to process their results	• Have the patients complete screeners in the lobby before their appointment (n=2).<br>• Have all educational handouts loaded into system for providers to easily print and give to patients to take home (n=2).

	Gaps Mentioned	Opportunities to Fill Gaps Mentioned
	6. Lower health literacy among patients (n=2)	• Nurses should ask questions twice to ensure the patient understands and close health literacy gaps (n=1).
	7. Anticipating a learning gap among clinic staff when new EHR system rolls out (learning gap may last 6-12 months before people are comfortable) (n=1)	• Practice using the new EHR system (n=1).
	8. Finding someone to fund efforts to make the screening process electronic (mobile or PC) for patients to fill out while they wait in the lobby (n=1)	None mentioned.
**Step 4: Follow-up procedures.**	1. Patient loss to follow up (n=9) due to people moving, changing numbers, or not recognizing GA CORE's area code during outreach (people think their call is unrelated to their clinic visit or a scam)	• Clinics could follow up with patients via phone and make sure they were contacted by Georgia CORE and connect them (n=5).<br>• Offering to schedule another visit before patient leaves the appointment (n=2).<br>• Send letters to patient homes (n=2).<br>• Increase education about follow-up on the patient's end and stress the importance of participating in follow up process (n=2).<br>• Letting patients know that the person following up may not be from the local clinic but rather GA CORE in Atlanta (giving area code information of the number that will be calling them) (n=2).<br>• Contacting patients using the local area code number that they would recognize (n=1).<br>• Make a note that the patient has not followed up with GA CORE in their patient file and nudge them to follow up during their next visit (n=1).<br>• Create an alert in the system that notifies you when patients try to reach out (n=1).<br>• Not passing over the calling process to nurses because they are already overburdened (n=1).<br>• Implementing reminder banners on the EHR systems to prompt nurses to screen patients (n=1).<br>• Send text reminders to patients to come in for screening (n=1).

	Gaps Mentioned	Opportunities to Fill Gaps Mentioned
	2. Unsure how to handle patients who do not want to be contacted regarding follow-ups (n=1)	None mentioned.
	3. Continue to provide education to patients who had a negative result as some patients think when they get a negative result that's it (n=1)	• Making sure the meaning of a negative result is thoroughly explained to the patient (n=1).
	4. When a patient hears they have a positive result, there may feel fear (n=2); may impact follow up	• Knowing how to handle discussing a positive result and being sensitive and assessing what barriers the patient may have to completing follow-ups (i.e. transportation, mental or emotional response to results) (n=1).<br>• Schedule a follow up appointment while the patient is in the clinic (n=1).<br>• Offer to connect them with GA CORE to learn more (n=1).
	5. Knowing what happened in the GA CORE referral process without having to read Cindy's letters, especially since different nurses may review patient files a year after the initial visit	• Updates to the EMR system that quickly let nurses know if a referral was made, if the patient received genetic testing, if they weren't responsive, etc. that can address loss to follow up (i.e., tickler system for PAP smear results) (n=1).
	6. Faxing is becoming obsolete -- need another mode to share patient results. They are also not a secure way of sharing health information (n=1)	• Result sharing feature/making referrals could be implemented into the EMR system (n=1).
