# Supplementary material for: A multilevel adaptation approach for the sustainable scale-up of family cancer history screening in rural public health clinics
Source: Arch Public Health. 2026 Jul 17;84:221. doi: 10.1186/s13690-026-02018-7 (PMC13660225; doi:10.1186/s13690-026-02018-7)
Supplement: Supplementary file 2 [file 13690_2026_2018_MOESM1_ESM.docx]

**Provider Interview Guide**

**INTERVIEW INTRODUCTION**

My name is XX. I am working as a research assistant on this research study at Emory University funded by the National Cancer Institute. The purpose of this study is to gather provider perspectives on your clinic’s approach to Family History Screening for Hereditary Breast and Ovarian Cancer. This interview will take about **one hour** to complete.

We will audiotape the interview. Be assured that your responses will be anonymized; your name and other identifying information will be removed from all audiotape transcripts.

Before we begin, I have a few questions to go over with you:

- Have you read the consent form? Do you have any questions about the study?
- Are you willing to take part? *[if no, stop the interview]*
- May I record this interview? *[if no, stop the interview]*

**START RECORDING**

**Family History (FH) Screening Workflow Diagram**

Our goal for the first set of questions is to understand how GA CORE’s family history (FH) screening program is implemented in **your** clinic's daily operations.

[START SHARING SCREEN]

Please look at this workflow diagram. Each **green** box represents a specific task performed by you or a staff member at your clinic. As we review each box, please describe the **standard procedures** associated with that step and specify which **staff member** in your clinic is typically responsible for its delivery.

**1. Schedule appointment:**

1) What type of patients access the Women’s Health services in your clinic?
2) On average, how many women do you see each week?
3) Who typically schedules appointments for Family History Screening?
4) Where does scheduling typically take place?
5) Is scheduling usually done online, via phone, or text message?
   a. Any reminder systems in place? Can you elaborate on what those systems entail (i.e. how frequently are reminders sent out, are reminders done online, via phone, text message, etc.?

2. **Complete check-in:** Please describe the standard process for patient check-in once patients arrive at the clinic.
   1) Who is responsible for completing check-in?
   2) Where does the check-in process usually take place?
   3) What is the average time spent on check-in process?
3. **Pre-FH screening activities:** Outline tasks performed by clinic staff before the FH screening. For each task, please describe who is responsible for completing the task, where, and the average time spent:
   1) Who?
   2) Where?
   3) Time spent?

[If no, proceed to the next section.]

4. **Administer FH screening:**
   1) Does your clinic have a protocol for administering the FH screening to patients?
   2) Who is responsible for administering the FH screening in your clinic?
   3) How many women are offered the FH screening per week?
a. What are the reasons that you do not administer the FH screening?
   4) How is this FH screening typically done?
a. Where?
b. When – e.g., before or after physical exams? at the beginning or end of the visit?
c. On a desktop or iPad?
d. When introducing the topic of FH screening to a woman, what do you say?
e. Do you use patient's zip code or the clinic's when completing family history?
   5) How long does it take to complete the FH screening?
a. On average, how much time does it take? The minimum time? The maximum time?
b. How often do you need to spend more time than average to complete FH? Why?
c. How often do you need to spend less time than average to complete FH? Why?

5. **Discuss FH screening results:**
   1) Does your clinic have a separate protocol for discussing the recommendations of the family history screening? Can you tell me a little more about that?
   2) For women who receive a **positive** FH screening result:
a. Who is responsible for discussing a positive screening result?
b. Can you walk me through specific language you use when discussing a positive FH screening result with a woman?
   i. What specific actions do you recommend to the patient?
   ii. Do you offer the Myriad education video during the visit? Why or why not?
   iii. Do you offer referrals to GA CORE during the visit? Why or why not?
   iv. Do you provide any other materials or resources?
c. From your experiences, how do women typically respond when receiving a positive FH screening result?
   i. Are there any recurring concerns or questions that women tend to raise?
d. Have you encountered any challenges when discussing positive FH screening results?
   i. time constraints
   ii. lack of private space
e. How long would you estimate that it takes to discuss a positive FH screening result?
   i. On average, how much time does it take? The minimum time? The maximum time?
   3) For women who receive a **negative** FH screening result:
a. Who is responsible for discussing a negative screening result?
b. Can you walk me through the specific language you use when discussing a negative FH screening result with a woman?
   i. What specific recommendations do you give the patient?
   ii. Do you provide any other materials or resources?
c. From your experiences, how do women typically respond when receiving a negative FH screening result?
   i. Are there any recurring concerns or questions that women tend to raise?
d. Have you encountered any challenges when discussing negative FH screening results?
   i. time constraints
   ii. lack of private space
e. How long does it take to discuss a negative FH screening result?
   i. On average, how much time does it take? The minimum time? The maximum time?
6. **Record FH screening results:**
   1) Who typically does this?
   2) Where?
   3) When? E.g., during or after the visit?
   4) What is the process for recording the FH screening results?
   5) Have you encountered any challenges when documenting the FH screening results?
   6) How long does it take to document the FH screening results? Average, min, max

7. **Follow-up procedures:**
   1) For **positive** results:
      a. Who makes the referral to GA CORE?
      b. When is the referral made? E.g., during or after the visit?
      c. Where does the referral process take place?
      d. What is the process for sending the referral to GA CORE?
      e. Who tracks the referral completion?
         i. How to track? Any reminder system?
         ii. How often to track?
      f. Have you encountered any challenges when making the referral?
      g. How long does it take to make the referral? Average, min, max
      h. Are there any other procedures?
   2) For **negative** results:
a. Are there any specific follow-ups for women with a negative FH screening result?
b. Who completes this task?
c. Where?

8. **Post genetic testing:** This next question is for patients who had a positive screening result and elected to undergo genetic testing. After a patient receives their genetic test result, what is the clinic's typical procedure or next steps?
   1) Who is responsible for following up with a patient after receiving their genetic testing results?

**Conclusion: That concludes the workflow diagram section of the interview.**

[STOP SHARING SCREEN]

**Barriers and facilitators to implementing the family history (FH) screening program.**

The next set of questions aim to understand how different factors might facilitate or hinder the implementation of FH screening in your clinic.

1. **Influence of Outer Setting on Inner Setting:**
   1) What are some external factors that may affect your ability to do FH screening in your clinic?
      i. Policies, availability of resources, community factors?
   2) Can you tell me about the characteristics of women you typically see in your clinic?
      i. How do you think this has shaped your clinic’s approach to conducting FH screening?
   3) Are there any groups within the Southwest GA community that you feel are underserved or overlooked when it comes to FH screening? Can you tell me more about that?
2. **Inner Setting Impacting the Process of Implementation:**
   1) How has your clinic’s culture facilitated or posed challenges to the implementation of FH screening? Please provide a specific example.
      a. Leadership or peer support for FH screening
   2) How does your clinic’s physical layout or other physical features influence the implementation process of FH screening?
      a. Private room
   3) What about technology infrastructure? How does this influence the implementation of FH screening?
      a. Can you send text message to patients?
      b. Is the FH screening done in patient portal?
   4) Have you been provided with training or resources to assist with FH screening implementation?
      a. How have the training and resources provided influenced your understanding/delivery of FH screening?
      b. Are there areas where further training or resources might be beneficial?
   5) Do you see any differences within your clinic regarding who gets FH screening and who doesn’t?
      a. Specific racial/ethnic groups? Distance to clinic? Insurance?
      b. Are there specific strategies that you are aware of that have been used in your clinic to reach these populations who may be experiencing challenges to access FH screening?
      c. For example, any community outreach strategies?
3. **Interplay Between Individual Characteristics and Implementation Process:**
   1) Do you have a designated individual or "champion" in your clinic responsible for implementing FH screening? How has this influenced your experiences implementing FH screening?
   2) Are there individuals in your clinic who may oppose the implementation of FH screening?
   3) How would you describe your level of knowledge (e.g., high, low) and beliefs about FH screening?
      a. How have these factors influenced your approach to communicate with patients about FH screening?
   4) Have you had situations where women declined the FH screening? What are the reasons?
4. **Sustainability Vision:**
1) Which core components of FH screening program do you think are absolutely essential for the consistent delivery of FH screening?
2) What procedures do you want to add to improve the program’s reach and effectiveness?
3) What procedures do you find redundant?
   a. Any overlapping responsibilities or tasks?
4) What procedures do you want to change? And how?
5. **Concluding Insights:** Are there additional recommendations or insights you’d like to share?

Thank you for your valuable input in today’s interview. Your insights are important for us to better understand FH screening practices at your clinic.

Next, we invite you to join a time study to delve deeper into your FH screening experiences. **You will record the time spent on three key FH screening tasks with patients over a month. This activity may require about 15 minutes of your time each week.** Here’s a preview of the timesheet **[showing on screen]**. You can choose between an electronic or paper version. I’ll send further instructions via email soon. Do you have any questions?

Once again, thank you for your time and participation. Our interview is now concluded.
